# Influence of depression symptoms on serum tumor necrosis factor-α of patients with chronic low back pain

**DOI:** 10.1186/ar3156

**Published:** 2010-10-11

**Authors:** Haili Wang, Carsten Ahrens, Winfried Rief, Simone Gantz, Marcus Schiltenwolf, Wiltrud Richter

**Affiliations:** 1 Department of Orthopaedic Surgery, University of Heidelberg, Schlierbacher Landstrasse 200a, 69118 Heidelberg, Germany; 2 Department of Clinical Psychology and Psychotherapy, University of Marburg, Gutenbergstrasse 18, 35032 Marburg, Germany

## Abstract

**Introduction:**

Patients with chronic low back pain (cLBP) have high rates of comorbid psychiatric disorders, mainly depression. Recent evidence suggests that depressive symptoms and pain, as interacting factors, have an effect on the circulating levels of inflammatory markers relevant to coronary artery disease. Our previous work showed a higher serum level of an inflammatory marker tumour necrosis factor-alpha (TNFα) in patients with cLBP, which did not correlate with intensity of low back pain alone. In the present study we investigated the cross-sectional associations of depressive symptoms, low back pain and their interaction with circulating levels of TNFα.

**Methods:**

Each group of 29 patients with cLBP alone or with both cLBP and depression was age-matched and sex-matched with 29 healthy controls. All subjects underwent a blood draw for the assessment of serum TNFα and completed a standardised questionnaire regarding medication, depression scores according to the German version of Centre for Epidemiological Studies Depression Scale (CES-D), pain intensity from a visual analogue scale, and back function using the Roland and Morris questionnaire. The correlations between TNFα level and these clinical parameters were analysed.

**Results:**

There were no differences in TNFα level between cLBP patients with and without depression. Both cLBP patients with (median = 2.51 pg/ml, *P *= 0.002) and without (median = 2.58 pg/ml, *P *= 0.004) depression showed significantly higher TNFα serum levels than healthy controls (median = 0 pg/ml). The pain intensity reported by both patient groups was similar, while the patients with depression had higher CES-D scores (*P *< 0.001) and worse back function (*P *< 0.001). The variance analysis showed that the interaction between TNFα level and pain intensity, CES-D scores, sex, body mass index and medication was statistically significant.

**Conclusions:**

Depression as a comorbidity to cLBP did not influence the serum TNFα level. It seems that TNFα somehow acts as a mediator in both cLBP and depression, involving similar mechanisms that will be interesting to follow in further studies.

## Introduction

Patients with chronic low back pain (cLBP) very often additionally present psychiatric disorders, mostly affective disturbance. The most common of these disorders is depression. Equally, depression is frequently associated with pain. Some 30 to 60% of cases of depression are accompanied by pain, and *vice versa *[1]. Pain is a major predictor of depression and anxiety, and depression seems to be an important predictor for work disability of patients with chronic pain [2]. The costs of medical treatment for cLBP patients with depression was found to be 2.8 times higher than those for patients without depression [3], and the economic cost of depressive disorders is higher in the presence of coexisting pain [4]. The accompanying depression should therefore be recognised early and taken into account in the treatment strategy for chronic pain [5-8].

To date, however, pain and depression have been considered as separate entities and therefore treated in isolation; the pathophysiology of both pain and depression are not yet wholly clarified. Patients with pain or depression have often been observed to present common clinical features such as lethargy, anorexia [9], sleepiness [10], hyperalgesia [11], reduction in grooming [12] and failure to concentrate [13], which indicates that pain and depression may share some aspects of pathophysiology represented by common pathways and neurotransmitters.

Over the past decade, numerous studies have demonstrated that cytokines seem to play an important role in both pain and depression, respectively [14-19]. Cytokines can, however, act as a central link between pain and depression [20]. This knowledge stimulates our interest in the potential involvement of immune impairment in coexisting cLBP and depression.

Our previous work revealed elevated TNFα serum level in patients with cLBP, and that confounding parameters such as age, sex, body mass index, alcohol, cigarettes, pain rating and back function did not influence the TNFα serum level [21]. This observation prompts the question: which factors did influence the TNFα serum level?

To date, no studies have investigated the TNFα profile in coexisting pain and depression. In the prospective cross-sectional clinical study described here, we set out to determine whether comorbid depression might affect the TNFα serum level in patients with low back pain, or whether TNFα might regulate both pain and depression together. Our hypothesis was that patients with both cLBP and depression display higher TNFα serum levels than patients with cLBP alone and there are cross-sectional associations of pain, depressive symptoms and their interaction with TNFα in cLBP.

## Materials and methods

### Subjects

All participants gave informed consent, and the study was approved by the local ethics committee of the University of Heidelberg, Germany. Participants were consecutively recruited from the Department of Orthopaedic Surgery of the University of Heidelberg. Each group of 29 patients with cLBP alone or with cLBP together with depression (cLBP + DE) were matched with 29 healthy controls by age and sex.

The inclusion criterion for pain was cLBP as the main symptom, defined as disabling pain of at least 6 months' duration that led to the patient being on sick leave for at least 6 weeks. Patients with other pain locations as their main symptom and patients with multiple major pain locations were excluded from this study.

The inclusion criteria for the diagnosis of depression were: an International Statistical Classification of Diseases and Related Health Problems (10th revision) diagnosis of a current and at least moderate depressive episode; and a minimum German version of Centre for Epidemiological Studies Depression Scale (CES-D) score of 25.

Exclusion criteria in patients and controls were: tumour disease (diagnosis from history and by radiographic examination/magnetic resonance imaging (MRI)); trauma/fracture (history and radiographic examination); inflammatory systemic disease or infection - for example, spondylodiscitis (blood count and radiographic evaluation/MRI); nucleus pulposus prolapse with corresponding radicular pain (clinical examination, MRI); structural pathology of the lumbar spine - for example, spinal stenosis or spondylolisthesis (radiographic evaluation/MRI and clinical examination); rheumatological disease; serious cardiopulmonary, vascular or other internal medical conditions; any sensorimotor and/or neurological deficits in the lower extremity (clinical examination); spinal surgery in the year before admission to multidisciplinary therapy; radiographically apparent degenerative changes in the lumbar spine (grade II or above according to the Kellgren and Lawrence classification [22]); or medication that may influence the TNFα level (for example, oral or local corticosteroids, aspirin, nonsteroidal anti-inflammatory drugs, anti-TNFα therapy).

### Evaluation

At study entry, the initial evaluation included clinical and radiographic examination and also MRI of the lumbar spine in all patients of the entire study, and blood count in all patients and controls. Patients were evaluated by standardised questionnaires and physical examinations, including analysis of blood samples.

The average pain intensity of all patients was determined from a visual analogue scale recording from 0 (no pain) to 10 (severe pain) during the past 24 hours and the past week. Measures of pain-related disability was assessed using the Roland and Morris questionnaire [23], which is a self-administered questionnaire consisting of 24 items chosen to reflect varied activities of daily living. An item receives a score of 1 if it is checked as applicable by the respondent, and a score of 0 if it is not marked. Accordingly, total scores can vary from 0 (no disability) to 24 (severe disability).

The CES-D is a well-established self-reporting instrument to assess the level of depression, with 20 items and a potential overall score of 0 to 60. It has high specificity (94%) for the identification of acute depression if a score of at least 23 points is reached and the correlation coefficient to other instruments for measuring depression, such as the Hamilton Depression Scale, is acceptable (*r *= 0.49) and increases with recovery from depression (*r *= 0.86) [24]. To identify other confounding factors, at each time point the patients filled in this standardised questionnaire about depression (CES-D), sleep duration, alcohol and nicotine consumption, and exercise. To identify confounding factors of medication, the drug intake in the two groups was studied accordingly to the Anatomical Therapeutic Chemical Classification System.

### Determination of cytokine levels in serum

At the given time points, venous blood was taken from the cubital vein between 8:00 and 9:00 am. Blood samples were centrifuged at 2,000 rpm at 4°C within 30 minutes of withdrawal, and serum was stored at -80°C. TNFα serum levels were analysed in duplicate using a Bio-Plex cytokine assay (Bio-Rad Laboratories, Munich, Germany) according to the manufacturer's instructions. The median fluorescence intensity of standards and patient samples were determined. Using the Bio-Plex Manager software, serum levels of TNFα were deduced from the standard curve. The intra-assay coefficient of variation was 5 to 10%.

### Statistical analysis

The nonparametric Mann-Whitney test was used to compare groups and was adjusted with Bonferroni correction. Correlations between the individual groups and cytokines were investigated using Pearson correlation analysis. Variance analysis was used to evaluate the interaction between TNFα level, pain intensity and depression scores. Drug intake was analysed as captured/noncaptured without considering the dose. *P *< 0.05 was considered statistically significant; *P *< 0.01 was highly significant. The data were analysed using SPSS 15.0 software (SPSS, Chicago, IL, USA).

## Results

### TNFα serum levels

**Figure 1:**
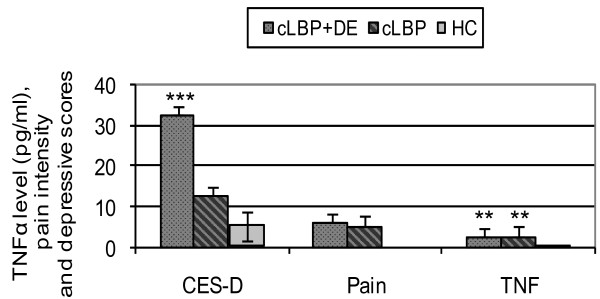
**TNFα serum levels**. Serum levels of proinflammatory cytokine TNFα (pg/ml, median), pain intensity and depression (DE) scores in patients with chronic low back pain (cLBP) and in healthy controls (HC). ***P < 0.001, **P < 0.01, differences between patient group and healthy controls. CES-D, Centre for Epidemiological Studies Depression Scale.

The circulating TNFα serum levels of patients with and without depressive symptoms were 2.51 pg/ml and 2.58 pg/ml (median), respectively (Figure 1). The levels were significantly higher than those of healthy controls (0.1 pg/ml) (*P *= 0.002 for cLBP + DE, *P *= 0.004 for cLBP). No differences in TNFα levels between the cLBP + DE group and the cLBP group were seen in this study.

### Clinical characteristics between groups

Table 1 presents the clinical characteristics of participants. Overall there were no statistically significant differences for age, sex, and body mass index between all three groups. The reported sleep duration, exercise level, or alcohol and nicotine consumption in the past 24 hours within the three groups were comparable (*P *> 0.05). Even the pain intensity of both patient groups did not differ from each other (*P *> 0.05). Patients with both cLBP and depressive symptoms, however, had significantly higher CES-D scores (*P *< 0.001) (Figure 1) and worse back function (*P *< 0.001) than patients with cLBP alone. The medication (inclusive antidepressants) intake between the two groups was contrastable, except that patients in the cLBP + DE group were taking significantly more nonsteroidal anti-inflammatory drugs (M01A) than patients with cLBP alone at T0 (*P *= 0.037).

**Table 1:** Clinical and psychosocial data for all subjects

	Healthy controls (*n *= 29)	cLBP + DE (*n *= 29)	cLBP (*n *= 29)
Sex (female/male)	17/12	17/12	17/12
Age (years)	40.72 (23 to 66)	45.31 (20 to 69)	44.69 (24 to 68)
Body mass index (kg/m^2^)	27.1 (18.7 to 47.8)	25.1 (18.9 to 37.4)	24.2 (17.7 to 33.3)
Sleep (hours)	6.7 ± 0.7	5.6 ± 1.7	6.4 ± 1.9
Pain 24 hours^a^	0	6.30 ± 1.89	5.13 ± 2.30
Pain 7 days^a^	0	6.29 ± 1.58	5.19 ± 2.27
CES-D score	5	32.81 ± 9.38*	12.68 ± 6.64
Back function (R&M)	0	13.77 ± 5.26*	10.19 ± 5.65
Exercise in past 24 hours (hours)	5.98 ± 2.33	5.68 ± 5.09	5.28 ± 3.95
Alcohol consumption (24 hours)	16%	16%	19%
Nicotine consumption (24 hours)	20%	42%	23%
Medication			
*Nonsteroidal anti-inflammatory drugs*	0	28%*	7%
*Opioids*	0	24%	28%
*Antidepressants*	0	24%	17%

Data presented as *n*, mean (range), mean ± standard deviation or percentage. cLBP, chronic low back pain; DE, depression; CES-D, Centre for Epidemiological Studies Depression Scale; R&M, Roland and Morris [23]. ^a ^: Visual analogue scale, 0 to 10. *: *P *< 0.05.

### Correlation between TNFα serum level and confounding factors

In an unadjusted analysis, no correlation was found between TNFα serum level and age, sex, body mass index, pain intensity, CES-D score or back function. The single correlation was found between the TNFα serum level and intake of analgesics in the cLBP + DE group (*P *= 0.027 > 0.005, *S *= -0.411), however, this single correlation disappeared after assessment of all variables (as there were 11 variables in this study, correlation should be *P *< 0.05/11 = 0.005).

In the variance analysis, using TNFα as a conditioned variable presented no significant interactions between TNFα, pain intensity in the past 24 hours (*P *= 1.000) and CES-D scores (*P *= 1.000).

## Discussion

Many separate studies have shown that the proinflammatory cytokine TNFα may play a role in the pathophysiology both of pain and depression. TNFα is responsible for the triggering of mechanical nociception [25], peripheral sensitisation of nociceptors [26] and central sensitisation of posterior horn neurons [27]. In a chronic constructive injury model, levels of TNFα and IL-6 were upregulated when the spinal nervous system was chronically injured [28-31]. Administration of TNFα inhibitor countered the associated pain behaviour and hyperalgesia [32,33]. The role of TNFα in pain was reported by many clinical studies of chronic pain [34], neuropathic pain [35,37] and fibromyalgia syndrome [38,39]. Concerning the role of TNFα in depression, a lot of studies demonstrated significantly higher TNFα levels in major depressive disorder patients compared with normal controls [40,44] and a decrease of these levels after treatment with antidepressants, then reaching similar levels to healthy controls [40].

A new observation from the current study is detection that the quantity of serum TNFα from patients with cLBP was not intensified with coexisting pain and depressive symptoms. Patients with both pain and depression or patients with pain alone showed no difference of circulating TNFα level. The interaction between TNFα level and pain intensity or depression scores was not statistically significant. It seems that the TNFα level related to depression or to pain may be regulated by the same mechanism. There are several plausible mechanisms that could explain these results and the relationship between pain, depression and TNFα.

Stresses could be a common source of inflammatory response in the body of both pain and depressive patients. Increased sympathetic nervous system activation in response to stress has been suggested to mediate inflammatory processes [45]. Conceivably, depression has been induced in many of our patients by stress - and chronic, persistent pain is the big stressor for the patients. Exposure to stressful life events such as bereavement, divorce and academic stress is reported to cause depression and impairments of cellular immune function that may affect each other [46-51]. Chronic stress impaired at least T-helper type 1 responses, including the TNFα response [49,52]. Stressful life events coincide with depressive episodes but can also activate the immune/inflammatory system, leading to excess secretion of cytokines. In addition, there are occasional reports of decreased cytokine secretion in conjunction with the administration of antidepressant medication [43,53-55].

Recent developments in neuroscience and psychoimmunology point to the coexistence of pain and depression. A newly published review pointed out that the pathophysiologies of pain and depression may overlap in many respects [56]. Several brain regions are implicated in both major depressive disorder and pain. For example, the insular cortex [57,58], the prefrontal cortex [59,60], the anterior cingulate cortex [61,62], the amygdala and the hippocampus [63-65] are activated and/or altered in response to both depression and pain. Moreover, Robinson [56] verified that shared neurocircuits and neurochemicals play an important role connecting the pathophysiologies of depression and pain disorders.

This knowledge parallels our opinion. We challenge the widely held cytokine hypothesis of both cLBP and depression with the alteration of proinflammatory cytokine TNFα.

The common feature between pain and depression in relation to cytokines is the stress reaction within the hypothalamic-pituitary-adrenal (HPA) axis that exists in patients with cLBP and in those with depression. In animals, stress can activate proinflammatory pathways in the brain by activation of microglial cells [66,67]. In humans, modulation of the immune system by stress is well known [68]. Studies of chronic and acute stress in models of human stress have shown higher circulating levels of IL-6 and TNFα than in controls [68-70]. In general, depressed patients have an activated HPA axis, increased levels of cortisol and increased circulating levels of several proinflammatory cytokines, which can further stimulate the HPA axis and cortisol production [71].

In individuals with depression there is evidence for malfunction of cortisol receptors leading to cytokine-induced cortisol resistance, impaired feedback inhibition of the HPA axis and sustained activation of immune cells [72,73]. Depression and psychological distress sensitise and enhance inflammatory responses to subsequent stressful events [68]. The release of proinflammatory cytokines by peripheral immune cells during inflammation, infection or trauma leads to release of proinflammatory cytokines by glia in the central nervous system; these cytokines are associated with induction and maintenance of pain [74]. Cytokines can enter the brain and cause alterations in the metabolism of serotonin and dopamine. Additionally, cytokines activate chronic renal failure, which in turn leads to an increase in serum glucocorticoid levels [75]. Under physiological conditions, increased serum glucocorticoid levels induce an inhibition of the HPA axis. After prolonged stress, this negative feedback mechanism is disrupted [56]. cLBP as a persistent stressor may possibly also interrupt the negative glucocorticoid feedback on the HPA axis.

Historically, pain and depression have been conceptualised and treated as discrete phenomena, although they are highly comorbid disorders. Our findings illustrate the importance of shared common neurochemicals (TNFα) in the development of both disorders, and may provide a suggestion for the physicians - who should carefully evaluate patients presenting with either cLBP or depressive symptoms and tailor their treatment accordingly.

Potential confounding factors for the TNFα serum level were analysed in the present study. Age, sex, body mass index, pain intensity, CES-D score and back function did not correlate with the TNFα serum level individually.

## Conclusions

The present study clearly shows that depression as a comorbidity did not influence the TNFα level in cLBP patients. In other words, a high TNFα level in patients with cLBP was not induced by accompanying depression. Our hypothesis was therefore not supported. Our previous work and the present study, taken together, confirm that there is no causal relationship between the TNFα serum level and cLBP, just as there is no causal relationship between cytokine alterations in the blood and depressive disorders. Rather, it seems that TNFα somehow acts as a mediator in both cLBP and depression, by similar mechanisms. In the further study, we followed the development both of pain intensity and depression scores parallel to the TNFα level in a longitudinal design of 6 months, and tried to determine the potential interaction between TNFα, pain and depression.

## Abbreviations

CES-D: German version of Centre for Epidemiological Studies Depression Scale; cLBP: chronic low back pain; HPA: hypothalamic-pituitary-adrenal; IL: interleukin; MRI: magnetic resonance imaging; TNF: tumour necrosis factor.

## Competing interests

The authors declare that they have no competing interests.

## Authors' contributions

HW conceived the hypothesis for the manuscript, participated in data collection, wrote the first draft of the manuscript and had primary responsibility for the manuscript process. CA participated in data collection and performed the initial statistical analyses. SG interpreted the results of statistical analysis. WR participated in the interpretation of data, and contributed to and approved the final manuscript. MS conceived the study and participated in its design and helped to draft the manuscript. WR conceived the study and supervise its design. All authors read and approved the final manuscript.
